# The “criminal” artery of de Winter may be the left circumflex artery: A CARE-compliant case report

**DOI:** 10.1097/MD.0000000000020585

**Published:** 2020-06-12

**Authors:** Dongpu Shao, Na Yang, Shanshan Zhou, Qingyuan Cai, Rangrang Zhang, Qian Zhang, Zhaoyang Wei, Hang Li, Yang Zheng, Qian Tong, Zhiguo Zhang

**Affiliations:** Department of Cardiology, the First Hospital of Jilin University, Changchun, Jilin Province, China.

**Keywords:** de Winter, left circumflex artery, myocardial infarction

## Abstract

**Rationale:**

De Winter et al first described a new ST-segment elevation myocardial infarction (STEMI)-equivalent pattern associated with acute occlusion of the left anterior descending coronary artery (LAD). Studies show that this pattern has a positive predictive value of 95.2% to 100%. However, some cases of non-STEMI, caused by acute right coronary artery or LAD diagonal branch occlusion, have been reported, which exhibit electrocardiogram (ECG) changes similar to the de Winter pattern. Few cases of de Winter ECG pattern caused by left circumflex artery (LCX) stenosis have been reported.

**Patient concerns:**

A 57-year-old man was admitted to the emergency department 12 hours after suffering from oppressive chest pain and diaphoresis. The patient had a history of diabetes and smoking. An initial ECG showed atrial fibrillation, upsloping ST-segment depression at the J point, followed by peaked, positive T waves in leads V2 to V6 and slight ST-segment elevation in lead aVR, with poor R-wave progression. Coronary angiography showed tubular stenosis (up to 95%) of the proximal portion of the LCX.

**Diagnosis:**

LCX stenosis led to a diagnosis of non-STEMI.

**Interventions:**

Left coronary artery stenosis was successfully treated with angioplasty and insertion of a drug-eluting stent.

**Outcomes:**

The patient's chest pain resolved completely after stent implantation. No myocardial infarction occurred during the 6-month follow-up period.

**Lessons:**

De Winter ECG pattern cannot be presumed to be associated with LAD stenosis and 18-lead ECG is required to support the identification of the “criminal” artery and to rule out posterior myocardial infarction.

## 1 Introduction

In 2008, de Winter et al first described a novel, characteristic ST-segment elevation myocardial infarction (STEMI)-equivalent electrocardiogram (ECG) pattern associated with acute occlusion of the proximal left anterior descending coronary artery (LAD).^[1]^ Diagnostic studies show that this pattern has a positive predictive value of 95.2% to 100%.^[2]^ However, some reports have identified non-STEMI caused by the acute total occlusion of the right coronary artery or LAD diagonal branch, which exhibits ECG changes similar to the de Winter pattern.^[3,4]^ The case described here summarizes a case of de Winter ECG pattern caused by left circumflex artery (LCX) stenosis. The case report was approved by the Institutional Review Board of the Hospital of Jilin University. Informed consent was obtained from the patient.

## 2 Case presentation

**Figure 1:**
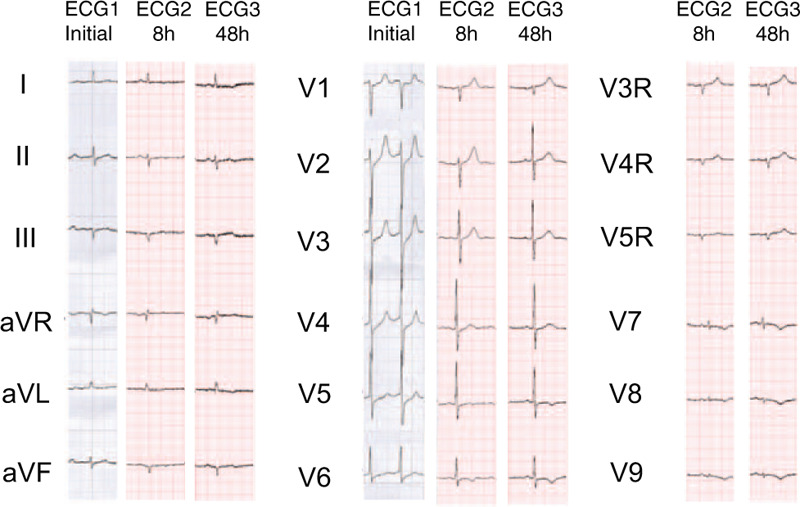
Initial 12-lead electrocardiogram (ECG 1) showing atrial fibrillation, upsloping ST-segment depression at the J point followed by tall, positive symmetrical T waves in leads V2 to V6 and slight ST-segment elevation in lead aVR, with poor R-wave progression. ECG 2 shows abnormal Q waves in leads III, aVF, and V9, and subsequent T-wave inversion in leads V5 to V9. ECG 3 shows no abnormal Q waves in lead V9.

A 57-year-old man was admitted to the emergency department 12 hours after suffering from oppressive chest pain and diaphoresis. The patient had a history of smoking and diabetes, although his blood glucose was not regularly monitored and treatment with oral metformin tablets was intermittent. An initial ECG obtained from a clinic when his pain had been persistent for 5 hours showed atrial fibrillation, upsloping ST-segment depression at the J point followed by peaked, positive T waves in leads V2 to V6 and slight ST-segment elevation in lead aVR, with poor R-wave progression (Fig. 1). Treatment with aspirin and clopidogrel was initiated immediately and the patient was transferred to the emergency department.

**Figure 2:**
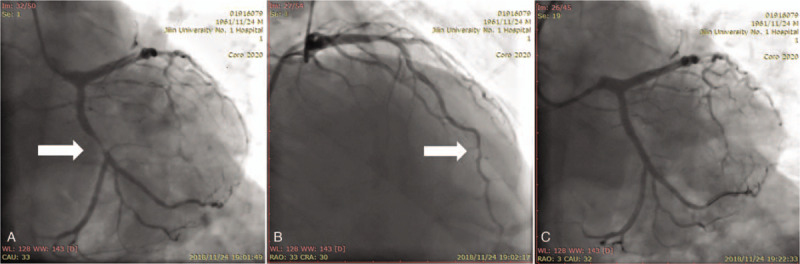
Coronary angiography showing: A. Tubular stenosis (up to 95%) of the proximal portion of the left circumflex artery; the advantage of coronary: mixed coronary dominance. The forward blood flow was thrombolysis in myocardial infarction grade 2 (arrow); B. Coronary angiography shows localized stenosis (approximately 60−70%) of the distal portion of the left anterior descending coronary artery (arrow). C. Blood flow was restored after percutaneous coronary intervention of the left circumflex artery.

At admission, the patient's blood pressure was 157/89 mm Hg, pulse rate was 78 beats/min and respiratory rate was 18 breaths/min on a non-rebreather mask, with a pulse oximetry reading of 96%. As lower intensity chest pain persisted, an 18-lead ECG was performed 8 hours after the initial ECG, showing sinus rhythm, abnormal Q waves in leads III, aVF, and V9, and T-wave inversion in leads V5 to V9 (Fig. 1). The vector of the ST-segment deviated towards the inferior and posterior wall of the left ventricle. Combined with the initial ECG findings, these changes were quickly identified by the emergency physician as de Winter syndrome (ECG changes showing no ST-segment elevation, upsloping ST-segment depression at the J point, and tall, symmetrical T waves in the precordial leads).^[1]^ Coronary angiography was performed immediately, showing localized stenosis (60%–70%) of the distal portion of the LAD and tubular stenosis (up to 95%) of the proximal portion of the LCX, which was successfully treated with angioplasty and the insertion of a drug-eluting stent (Fig. 2). The peak troponin I value was extremely high (52.8 ng/mL, reference range: 0−0.034 ng/mL).

The patient's chest pain resolved completely after stent implantation and a third ECG showed no abnormal Q waves in lead V9. Echocardiography performed 3 days after admission showed decreased amplitude of left ventricular inferior wall pulsation. These ECG and echocardiography changes indicate that the “criminal” artery in this case can be presumed to be the LCX. No myocardial infarction occurred during the 6-month follow-up period.

## 3 Discussion

De Winter et al first described a new STEMI-equivalent pattern of ECG changes associated with acute occlusion of the LAD.^[1]^ Cases of a de Winter ECG pattern caused by LCX stenosis have also been reported.^[5]^ The case reported here indicates that de Winter ECG pattern cannot be presumed to be associated with LAD stenosis and 18-lead ECG is required to identify the “criminal” artery. It is interesting to note that despite acute LCX sub-occlusion, ST-segment elevation was not seen during the entire duration of the myocardial infarction in this patient. The potential mechanisms of the ECG pattern remain unclear. As the peak troponin I value was extremely high in this patient, it is possible that the area of transmural ischemia was too large to generate injury currents toward the precordial leads. However, cardiovascular magnetic resonance imaging was not performed in this patient and the area of myocardial infarction was, therefore, unknown.

In comparison with patients exhibiting classical STEMI on ECG evaluation, those with a de Winter ECG pattern are generally younger, are more often men, and have a higher incidence of dyslipidemia.^[2]^ These characteristics may, therefore, help to quickly identify a de Winter ECG pattern. In many cases of LCX occlusion, ST elevation can only be detected in leads V7 to V9.^[5]^ However, 18-lead ECG is not routinely employed, meaning that many STEMI cases caused by LCX occlusion remain undiagnosed.^[6]^ We suggest that 18-lead ECG should be routinely performed to identify posterior wall myocardial infarction caused by LCX occlusion, particularly when the ECG shows ST depression primarily in leads V2 to V3.

## Acknowledgments

We thank the patient for the trust and agreement.

## Author contributions

**Data curation:** Dongpu Shao, Shanshan Zhou, Zhiguo Zhang.

**Investigation:** Dongpu Shao, Qingyuan Cai, Rangrang Zhang, Qian Zhang, Zhaoyang Wei, Zhiguo Zhang.

**Supervision:** Hang Li, Yang Zheng, Qian Tong, Zhiguo Zhang.

**Writing – original draft:** Dongpu Shao, Zhiguo Zhang.

**Writing – review & editing:** Na Yang, Zhiguo Zhang.
